# Potential of combination of DCE-MRI and DWI with serum CA125 and CA199 in evaluating effectiveness of neoadjuvant chemotherapy in breast cancer

**DOI:** 10.1186/s12957-021-02398-w

**Published:** 2021-09-18

**Authors:** Jun Zhang, Yongbo Huang, Jianghui Chen, Xia Wang, Hongyu Ma

**Affiliations:** 1 Radiological Department, Gaomi People’s Hospital, Gaomi, 261500 Shandong Province China; 2 CT Radiology, Gaomi People’s Hospital, Gaomi, 261500 Shandong Province China; 3 Department of Electrical and Computer Engineering, National University of Singapore, Singapore, 117583 Singapore

**Keywords:** Breast cancer, CA125, CA199, DCE-MRI, DWI, Neoadjuvant chemotherapy

## Abstract

**Background:**

To determine the potential of the combination of DCE-MRI imaging method with DWI and serum CA125 and CA199 levels in the evaluation of the efficacy of neoadjuvant chemotherapy in breast cancer patients.

**Methods:**

Sixty-five breast cancer patients who received neoadjuvant chemotherapy in our hospital from April 2016 to April 2017 were selected as research subjects. The patients received 4 courses of neoadjuvant chemotherapy. Lesions were monitored using DCE-MRI and DWI, while ELISA was used to measure the serum expression levels of the tumour markers CA125 and CA199. The patients were divided into the remission group and ineffective group based on pathological diagnosis.

**Results:**

There were significant differences in K_ep_, K_trans_, ADC_min_, ADC_mean_, tumour volume, and serum levels of CA125 and CA199 in patients in the remission group, before and after neoadjuvant chemotherapy, and there were significant differences in post-chemotherapy values of these indexes between the remission group and the ineffective group (*p* < 0.01).

**Conclusion:**

Combination of DCE-MRI diagnostic imaging with DWI can directly reflect the lesions in breast cancer patients after neoadjuvant chemotherapy. Serum levels of CA125 and CA199 levels are useful for evaluation of the impact of neoadjuvant chemotherapy on breast cancer patients, including risk of cancer cell metastasis and changes in some small lesions.

## Introduction

Breast cancer is one of the common gynecological diseases with high morbidity and mortality, and it is a serious threat to the health of women [1–3]. Neoadjuvant chemotherapy refers to systemic chemotherapy before surgical resection or local radiotherapy on the lesions. It reduces the risk of cancer cell metastasis to a certain extent, thereby facilitating follow-up intervention to control the disease conditions of the patients [4–6]. Based on years of clinical experience, neoadjuvant chemotherapy is often used in clinical intervention in patients with tumours [7–9]. With advancements in technology, the conventional MRI imaging method and measurement of levels of tumour markers are also used for evaluating the therapeutic effect of neoadjuvant chemotherapy on breast cancer [10, 11]. In this study, the potential value of neoadjuvant chemotherapy in the evaluation of the effect of neoadjuvant chemotherapy in breast cancer was evaluated using the diagnostic imaging method of DCE-MRI and DWI in combination with serum levels of CA125 and CA199. The study was aimed at providing useful information for the improvement of clinical diagnosis and treatment of breast cancer.

## Methods

### General information on subjects

This was a prospective study on 65 breast cancer patients who received neoadjuvant chemotherapy in our hospital from April 2016 to April 2017. Table 1 shows the general information on the patients.

**Table 1:** General information of patients

Parameters		Data
Number of patients		65
Age (years old)		35-55
Average age (years old)		46.6±9.1
Height (cm)		163.3±5.6
Weight (kg)		53±5.3
Body mass index (kg/m^2^)		25.6±3.9
TNM staging (number of patients)	T1 T2 T3	13 33 19

### Inclusion and exclusion criteria

#### Inclusion criteria

The included patients were those who had a detailed understanding of the aim of the study and signed informed content to participate in it, as well as patients who had no contraindication to imaging examinations used. Moreover, patients who had no other malignant tumours, and patients who accepted neoadjuvant treatment, were included in the study.

#### Exclusion criteria

Patients whose clinical data were incomplete, and patients whose conditions were complicated with inflammation and other diseases which might have a serious influence on the results of this study, were excluded. Moreover, patients who did not cooperate with clinical follow-up were excluded.

### Neoadjuvant chemotherapy regimens

The patients received chemotherapy with CEF (or CTF) regimen. For this purpose, the patients were treated with epirubicin, cyclophosphamide and docetaxel at doses of 600, 60 and 75 mg/m^2^, respectively (within the same day) via an intravenous drip, once a week, with 3 weeks as one complete course. In all, the patients received four courses of chemotherapy.

### Parameters measured

#### Assay of serum markers

Serum expression levels of CA125 and CA199 in patients were assayed using ELISA. Fasting venous blood (5 mL) collected from each patient was taken up in a sterile centrifuge tube. The blood samples were heated in a water bath at 37 °C and centrifuged at 3500 rpm for 3 min to obtain serum samples. The serum expression levels of CA125 and CA199 were assayed using ELISA kits (Abcam, UK).

The GE Signa HDxt1.5 T scanner (Car-Escorting Corporation, Chicago, USA) with a phase-controlled coil of 8 channels was used to carry out DWI and DCE-MRI on the patients.

#### Measurement of DCE-MRI

The conditions used were sequence of T1WI-VIBE, TR of 5000 ms, TE of 30 ms, FOV of 30× 30 cm^2^, pixel of 0.5 mm× 0.5 mm× 3.0 mm, NEX of two times, layer thickness of 3.0 mm, and layer spacing of 1.0 mm. After obtaining the mask, channels for intravenous injection were established on the back of the hand of each patient and Gd-DTPA was used as a contrast agent, with an injection dose of 0.2 mmol/kg and injection rate of 2.5 mL/s. Thereafter, DCE-MRI was immediately performed, and the dynamic enhanced images were transferred to TISSUE 4D software to determine the location of tumours. The average value of lesions was taken after three measurements, and the pseudo-colour images were generated. Then, the following quantitative parameters were calculated:

1. Volume transfer constant (K_trans_) was the velocity constant of intravascular contrast agent diffusing outside the blood vessels.
2. Rate constant (K_ep_) was the velocity constant of extravascular contrast agent penetrating into the blood vessels.
3. Volume fraction of extravascular extracellular space (*V*_*e*_): This was calculated as shown below:

$$ {V}_e:\frac{\left(\mathrm{Vascular}\ \mathrm{volume}+\mathrm{extracellular}\ \mathrm{space}\ \mathrm{volume}\right)}{\mathrm{Total}\ \mathrm{volume}} $$

#### Measurement of DWI

The patients were scanned using single-shot echo planar imaging technique, with SE-EPI sequence, under the following conditions: TR of 300 ms, TE of 86.0 ms, acquisition matrix of 320 × 320, FOV of 35, 18 layers, layer thickness of 4.0 mm, layer spacing of 1.0 mm and NEX of 6 times. The ADC values were calculated under gradient factor b value at 0 or 1000s/mm^2^.

### Assessment of effectiveness of neoadjuvant chemotherapy in the two groups of patients

The patients were divided into the remission group and ineffective group, based on the pathological diagnosis. The quantitative parameters for DCE-MRI (K_ep_, *V*_*e*_ and K_trans_); DWI apparent diffusion coefficients (ADC_min_ and ADC_mean_), tumour size, and serum expression levels of CA125 and CA199 were compared between the two groups before and after chemotherapy.

### Statistical analysis

The calculation of sample size and analysis of data obtained in this study were carried out using the SPSS version 20.0 software (International Business Machines Corporation, NY, USA). Count data were statistically analysed using *χ*^2^ test, while measurement data were analysed with Student’s *t* test. Differences were assumed statistically significant at *p* < 0.05.

## Results

### Comparison of DCE-MRI quantitative parameters before and after neoadjuvant chemotherapy

Table 2 shows a comparison of DCB-MRI quantitative parameters before and after neoadjuvant chemotherapy between the remission group and the ineffective group. There were no significant differences in K_ep_, K_trans_ and V_e_ between the two groups before neoadjuvant chemotherapy *(p>* 0.05). However, there were significant changes in K_ep_ and K_trans_ in the remission group after intervention, and there were significant differences in values of K_ep_ and K_trans_ between the remission group and the ineffective group after neoadjuvant chemotherapy (*p* < 0.01).

**Table 2:** Comparison of DCE-MRI quantitative parameters before and after neoadjuvant chemotherapy

Indexes	Group	Number of cases	Before chemotherapy	After chemotherapy	*t*	*P*
K_ep_ (/min)	Remission group	39	1.79±0.5	0.79±0.27	9.32	<0.01
	Ineffective group	26	1.67±0.53	1.51±0.53	1.19	>0.05
	*t*		0.93	7.21		
	*P*		>0.05	<0.01		
K_trans_ (/min)	Remission group	39	1.39±0.51	0.67±0.29		
	Ineffective group	26	1.27±0.51	1.13±0.33	6.52	<0.01
	*t*		0.93	5.93	1.24	>0.05
	*P*		0.36	0.00		
	Remission group *V*_*e*_	39	0.75±0.31	0.77±0.19	0.29	<0.01
	Ineffective group *V*_*e*_	26	0.83±0.33	0.85±0.13	0.29	>0.05
	*t*		0.99	1.87		
	*P*		>0.05	>0.05		

### Comparison of DWI results before and after neoadjuvant chemotherapy

Comparison of DWI results in the remission group and the ineffective group before and after neoadjuvant chemotherapy is shown in Table 3. Before chemotherapy, there were no significant differences in ADC_min_ and ADC_mean_ between the two groups (*p* > 0.05). However, there were significant differences in values of ADC_min_ and ADC_mean_ in the remission group before and after intervention, and there were significant differences in the values of these parameters between the remission group and the ineffective group after chemotherapy *(p <* 0.01).

**Table 3:** Comparison of DWI results before and after neoadjuvant chemotherapy

Indexes	Group	Number of cases	Before chemotherapy	After chemotherapy	*t*	*P*
ADC_min_	Remission	39	0.72±0.16	1.39±0.21	15.85	<0.01
	Ineffective	26	0.69±0.13	0.79±0.19	2.21	<0.05
	*t*		0.80	11.71		
	*P*		>0.05	<0.01		
ADC_mean_	Remission	39	0.77±0.19	1.46±0.23	14.44	<0.01
	Ineffective	26	0.74±0.17	0.87±0.13	3.10	<0.01
	*t*		0.65	11.86		
	*P*		>0.05	<0.01		

### Tumour volume before and after neoadjuvant chemotherapy

**Fig. 1:**
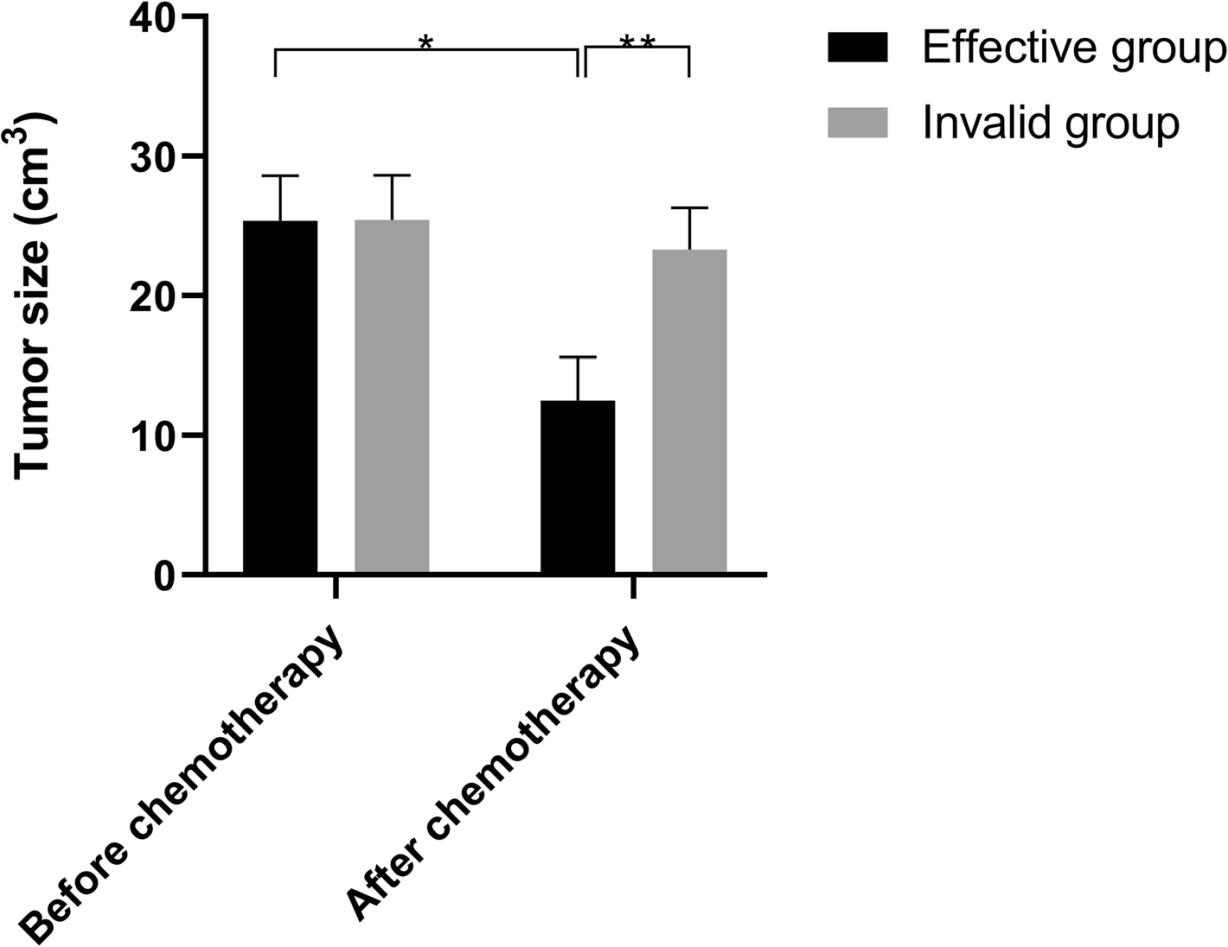
Tumour volume in the two groups before and after neoadjuvant chemotherapy. *P < 0.01, tumour volume of patients in the remission group before neoadjuvant chemotherapy (25.39±3.21cm^3^) vs tumour volume after neoadjuvant chemotherapy (12.51±3.09 cm^3^); **P < 0.01, tumour volume of patients in remission group (25.39±3.21 cm^3^) vs tumour volume of patients in ineffective group (23.32±3.01 cm3) after neoadjuvant chemotherapy

Figure 1 shows tumour volumes in patients before and after intervention. There was no significant difference in tumour volume of patients in the ineffective group before and after treatment (*p* > 0.05). However, after neoadjuvant chemotherapy, tumour volume of the remission group was significantly reduced, and there was a significant difference in the tumour volume between the two groups after neoadjuvant chemotherapy (*p* < 0.01).

### Serum CA125 levels of patients before and after neoadjuvant chemotherapy

**Fig. 2:**
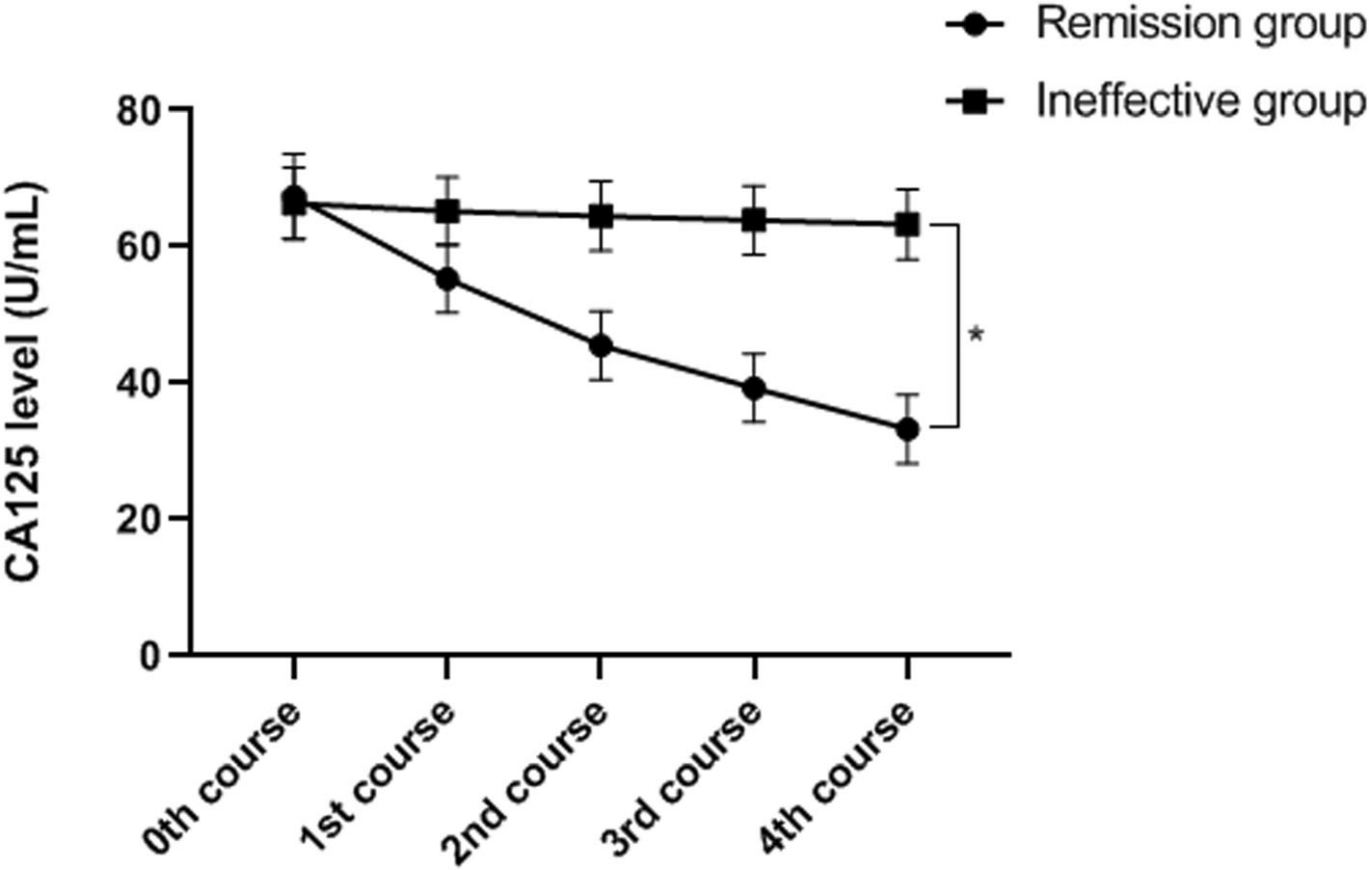
Serum CA125 levels of patients before and after neoadjuvant chemotherapy. *P < 0.01, serum CA125 level between the remission group (33.27±5.03 U/mL) and ineffective group (63.27±5.13 U/mL after 4 courses of neoadjuvant chemotherapy

Figure 2 shows serum CA125 levels of patients before and after neoadjuvant chemotherapy. Before treatment, there was no significant difference in CA125 level between the two groups (*p* > 0.05). In contrast, after neoadjuvant chemotherapy, the CA125 level in the remission group was significantly lower than the corresponding level in the ineffective group (*p* < 0.01).

### Serum CA199 levels in patients before and after neoadjuvant chemotherapy

**Fig. 3:**
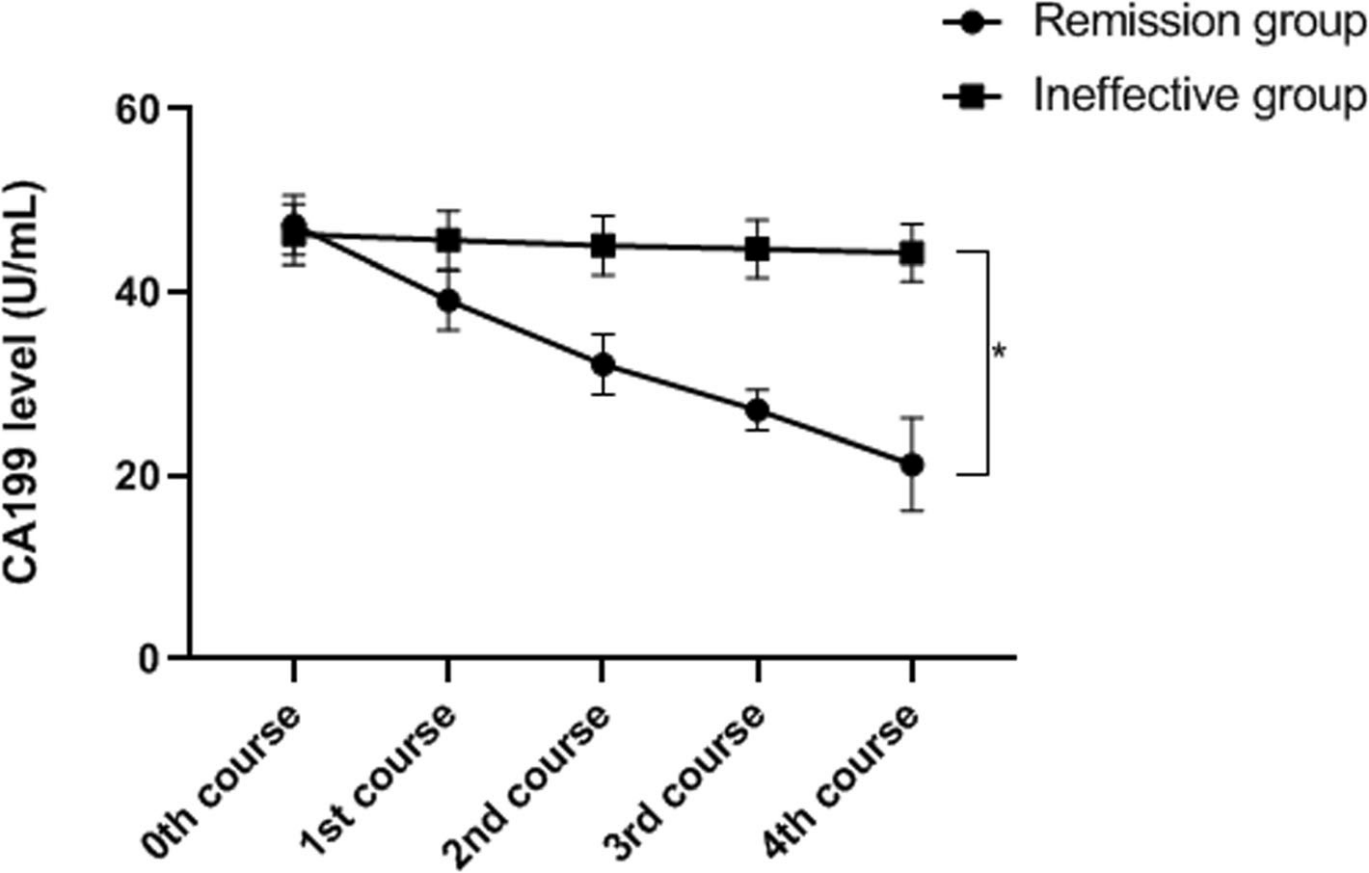
Serum CA199 levels of patients before and after neoadjuvant chemotherapy. *P < 0.01, comparison of serum CA199 level of patients (after 4 courses of neoadjuvant chemotherapy) between the remission group (21.23±5.03 U/mL) and the ineffective group (44.29±3.13 U/mL)

The serum CA199 levels in patients before and after neoadjuvant chemotherapy are presented in Fig. 3. Before treatment, there was no significant difference in CA199 level between the two groups *(p >* 0.05). However, after neoadjuvant chemotherapy, CA199 level in the remission group was markedly lower than that in the ineffective group (*p* < 0.01).

## Discussion

Neoadjuvant chemotherapy is often used as an adjuvant intervention in the clinical treatment of breast cancer patients [12, 13]. Before tumour resection or local chemoradiotherapy, the use of neoadjuvant chemotherapy reduces the lesion volume to a certain extent, and prevents the spread of cancer cells, resulting in a positive effect on subsequent treatments [14, 15]. However, there is a need for more effective methods for the evaluation of patient responses to neoadjuvant chemotherapy. With advances in the development of imaging diagnostic technology, the accuracy of DCE-MRI and DWI in tumour diagnosis has been continuously improved. Thus, these techniques have been gradually applied in the evaluation of the efficacy of neoadjuvant chemotherapy. The imaging function of DWI directly reflects the lesions in patients based on changes in diffusion of water molecules in the lesions [16]. Studies have shown that the higher the density of pathological cells in tumours, the higher the degree of limitation to the diffusion of internal water molecules. The ADC value obtained by DWI detection can accurately quantify the diffusion value of internal water molecules in the tissue, thereby effectively revealing the degree of tumour lesion in the patient [17]. This study has shown that the results of DCE-MRI and DWI in patients in the remission group after neoadjuvant chemotherapy were significantly different from those before intervention, and they were significantly different from those in the ineffective group. Thus, DCE-MRI and DWI can be reasonably applied for the evaluation of the results of neoadjuvant chemotherapy. Neoadjuvant chemotherapy is a globally-used intervention on cancer patients before tumour resection or targeted therapy [18, 19]. Although diagnostic imaging can directly reflect changes in lesions in patients, it is often unable to effectively determine cancer cell metastasis and small pathological tissues.

Tahmassebi et al. [20] have reported that diagnosis with DCE-MRI and DWI was effective for evaluation of the therapeutic effect of neoadjuvant chemotherapy in breast cancer patients. Studies have shown that CA15-3, CEA, CA125 and CA199 and other cancer-related factors, to a large extent, reflect the state of tumours, which is of great significance in the diagnosis, intervention and clinical follow-up of breast cancer patients [21, 22]. The serum expression levels of CA125 and CA199 in breast cancer patients are usually significantly higher than those in healthy people [22, 23]. This study showed that the serum levels of CA125 and CA199 in patients in the remission group were significantly reduced during neoadjuvant chemotherapy, and there were significant differences in the serum CA125 and CA199 levels after neoadjuvant chemotherapy between the remission group and the ineffective group. These results indicate that the serum expression levels of CA125 and CA199 can effectively be used to determine the therapeutic effect of neoadjuvant chemotherapy. Although serum CA125 and CA199 levels reflect the progression of breast cancer to an extent, they do not directly reflect the lesions in patients. Moreover, these indexes are associated with some limitations when used to guide subsequent treatments and interventions. In addition, a study has shown that there were significant changes in the serum CA125 levels of patients before and after neoadjuvant chemotherapy [24]. This is consistent with the results of the present study.

## Conclusion

The present study has demonstrated that the imaging diagnostic technique of DCE-MRI and DWI are effective in reflecting changes in lesions in breast cancer patients after neoadjuvant therapy. Moreover, the study has shown that serum levels of CA125 and CA199 reflect the impact of neoadjuvant chemotherapy on patients with respect to risk of cancer cell metastasis and changes in some small lesions. These findings indicate that DCE-MRI and DWI, in combination with serum tumour factors CA125 and CA199 can provide more comprehensive and accurate guidance for subsequent clinical treatments of breast cancer patients.

## Data Availability

The datasets used and/or analysed during the current study are available from the corresponding author on reasonable request.
